# Revised and annotated checklist of aquatic and semi-aquatic Heteroptera of Hungary with comments on biodiversity patterns

**DOI:** 10.3897/zookeys.501.8964

**Published:** 2015-04-30

**Authors:** Pál Boda, Tamás Bozóki, Tamás Vásárhelyi, Gábor Bakonyi, Gábor Várbíró

**Affiliations:** 1 MTA Centre for Ecological Research, Department of Tisza River Research, Bem tér 18/c., H-4026 Debrecen, Hungary; 2 Eszterházy Károly College, Eszterházy tér 1., H‐3300, Eger, Hungary; 3 Hungarian Natural History Museum, Baross u. 13., H-1088, Budapest, Hungary; 4 Szent István University, Department of Zoology and Animal Ecology, Páter Károly u. 1., H-2100, Gödöllő, Hungary

**Keywords:** Water bugs, estimated species richness, new species records, *Notonecta reuteri reuteri*

## Abstract

A basic knowledge of regional faunas is necessary to follow the changes in macroinvertebrate communities caused by environmental influences and climatic trends in the future. We collected all the available data on water bugs in Hungary using an inventory method, a UTM grid based database was built, and Jackknife richness estimates and species accumulation curves were calculated. Fauna compositions were compared among Central-European states. As a result, an updated and annotated checklist for Hungary is provided, containing 58 species in 21 genera and 12 families. A total 66.8% of the total UTM 10 × 10 km squares in Hungary possess faunistic data for water bugs. The species number in grid cells numbered from 0 to 42, and their diversity patterns showed heterogeneity. The estimated species number of 58 is equal to the actual number of species known from the country. The asymptotic shape of the accumulative species curve predicts that additional sampling efforts will not increase the number of species currently known from Hungary. These results suggest that the number of species in the country was estimated correctly and that the species accumulation curve levels off at an asymptotic value. Thus a considerable increase in species richness is not expected in the future. Even with the species composition changing the chance of species turn-over does exist. Overall, 36.7% of the European water bug species were found in Hungary. The differences in faunal composition between Hungary and its surrounding countries were caused by the rare or unique species, whereas 33 species are common in the faunas of the eight countries. Species richness does show a correlation with latitude, and similar species compositions were observed in the countries along the same latitude. The species list and the UTM-based database are now up-to-date for Hungary, and it will provide a basis for future studies of distributional and biodiversity patterns, biogeography, relative abundance and frequency of occurrences important in community ecology, or the determination of conservation status.

## Introduction

Aquatic and semi-aquatic Heteroptera (water bugs) are important components of aquatic ecosystems for several reasons. Water bugs act both as consumers of algae and leaf litter at lower trophic levels and as prey for fish and other organisms at higher trophic levels (McCafferty 1981, Hutchinson 1993). Water bugs can be found on the macrophyte stands, of the benthic region, beneath open water or on the surface. However, both the surface dwellers and the truly aquatic forms occupy a particular niche within an ecosystem (Savage 1989). Moreover, several species are considered as flagship or umbrella species for ecosystem protection (Whiteman and Sites 2008). In addition to their ecological role, some species even have high economic importance as top predators or food sources for protected or endangered animals (or even humans), the significance of which has probably been underestimated (Papáček 2001).

There are conflicting opinions in the literature as to whether aquatic bugs are good indicators of the ecological status. However, communities of aquatic Heteroptera *per se* have generally been studied less frequently than the assemblages of aquatic macroinvertebrates as a whole (Turić et al. 2011). Aquatic bugs – except for nymphs and *Aphelocheirus aestivalis* – are air-breathers, thus, they exist under a wide range of water quality conditions, including waters poor in oxygen. On the other hand, the distribution of some taxa is correlated with several biotic and abiotic factors (e.g., Macan 1938, 1954, Savage 1982, Tully et al. 1991, Savage 1994, Sládeček and Sládečková 1994, Hufnagel et al. 1999, Jardine et al. 2005, Nosek et al. 2007). Consequently, some aquatic bugs show great sensitivity to environmental stressors, whereas some other species are more resilient to environmental changes which, on the whole makes them doubtful indicators of water quality. This ecological difference may be related to their geographic distribution. Due to their high dispersal ability, some species with a wide ecological tolerance to environmental constraints can be found in almost every freshwater habitat across the Holarctic Region. Besides these cosmopolitan taxa, there are species which occur exclusively in specific habitats (Macan 1954, Savage 1994).

The aquatic and semi-aquatic Heteroptera are composed of two monophyletic infraorders (Gerromorpha, Nepomorpha), which together encompass 92% of the aquatic and semi-aquatic species, with the remaining species belonging to the more or less water dependent Leptopodomorpha (Polhemus and Polhemus 2008). In the Palaearctic Region, there are more than 100 Gerromorpha and 200 Nepomorpha species. The first major catalogue of species in the Palaearctic Region was published by Aukema and Rieger (1995), who presented all the synonyms and distribution information based on the original descriptions. This work was later supplemented and up-dated by Aukema et al. (2013). In Hungary, active taxonomical and faunistical studies have been conducted since 1870. The first Heteroptera checklist, encompassing both terrestrial and aquatic species, was published by Horváth (1918), and since then, Hungarian experts have published almost 100 publications containing faunistic data on aquatic bugs. The large amount of relevant new information was summarised in a new checklist by Kondorosy (1999). Since 1999, attention focused mainly on the autecology of aquatic and semi-aquatic Heteroptera and has remained relevant since the EU Water Framework Directive (WFD) was adopted (European Commission 2000). The WFD, undoubtedly, represents a milestone in the research of the aquatic and semi-aquatic Heteroptera fauna of Hungary, and the member states of the European Union.

The implementation of the WFD required intensive faunistical and ecological surveys across Hungary. The first country-wide survey of aquatic and semi-aquatic Heteroptera was carried out in 2005 under the framework of the ECOSURV project (Kiss et al. 2006a,b). The increasing intensity of faunistic research is clearly illustrated by the fact that more papers were published during the last 15 years (*N* = 103, 1999-2014) than in the previous decades (*N* = 95, prior to 1999). Many localities that had been poorly studied before were sampled and five heteropteran species new to the Hungarian fauna have been detected since 1999. Consequently, this large amount of new data warrants a comprehensive faunistical overview of this group.

The main goals of the present paper are (1) to provide a revised and annotated checklist of the aquatic and semi-aquatic Heteroptera fauna of Hungary, (2) to assess the UTM-based distributional patterns during three distinct intervals of research to show the biodiversity trends in Hungary over more than 100 years, (3) to describe the current state-of-the-art of water bug studies in Hungary, and (4) to compare the number of species with those of the neighbouring countries. Finally, by synthesizing this information, key areas for future research are identified.

## Material and methods

### Geographic and hydrological background

Hungary is located in the Carpathian Basin, the largest intramontane basin in Europe (Gábris and Nádor 2007). Most of the country lies below 200 m a.s.l.; the highest point in the country is Kékes (1 014 m) and the lowest spot is located near Szeged in the south (77.6 m). Based on the ecoregion classification schemes of rivers and lakes (EEA 2004, Illies 1978), Hungary belongs to the Pannonian Ecoregion. This alluvial basin is formed by the Danube River and its main tributaries, the Tisza and Dráva Rivers. The hydrology of Hungary is primarily determined by these large potamal rivers. The most characteristic water body types are the small lowland streams, oxbows, swamps, and soda pans formed by fluvial erosion and deflation (Borics et al. 2014). Besides these types of waters, large, shallow lakes (e.g., Lake Balaton, Lake Velence and Fertő) provide unique habitats for aquatic and semi-aquatic Heteroptera in Hungary.

### Database, statistical analyses

As a first step, a database was constructed that contained information on the taxa occurring in Hungary and their known locations. During the building of the database, two main sources were considered: published papers, and data from the regular surveillance monitoring operated by the National Environmental Authorities since 2005. As a result, 22 587 records from 198 papers published between 1878 and 2014 are included in the database. Records were only included when the specimen was identified to species and when the locality of occurrence was clearly indicated. For mapping the distribution patterns, all records were arranged into 10 × 10 km UTM grids. Non-verifiable records were omitted from the database. To reveal the trends in the growth of knowledge regarding water bugs, the database was divided into three time periods: the first part included all records before 1918, the second part included all records before 1999, and the third part contained all data before 2014, respectively. Each sub-database was then considered as a matrix with UTM grids in columns and species in rows. Each species has presence-absence data in cells appertaining only to those UTM grid cells, in which aquatic and semi-aquatic Heteroptera data occurred during the given period. Based on these sub-databases, species accumulation curves and richness estimates were calculated with PAST 3.02 (Hammer et al. 2001). Jackknife 1 was used as a non-parametric estimator, because it is useful for evaluating the expected richness for incidence data (Melo 2004, Gotelli and Colwell 2010).

The composition of water bug assemblages of the neighbouring countries were compared using non-metric multidimensional scaling (NMDS). The dissimilarity of assemblages based on presence-absence data was quantified by the Jaccard index (Legendre and Legendre 1998). The correlation between the number of species and the number of UTM grids was also calculated with PAST 3.02.

### Compiling the checklist

The names of the species were updated according to Aukema and Rieger (1995) and Aukema et al. (2013). A detailed taxonomic classification is listed in the current checklist, with the author of each taxonomy level given. New records were identified by the authors Kiss et al. (2009), Soós et al. (2009), and Soós et al. (2010). All of the changes between the second and the latest checklist (Kondorosy 1999) were noted, and finally we produced an updated checklist of Hungarian aquatic and semi-aquatic Heteroptera. Following Nieser (2002), we considered the subfamily Micronectinae to have family rank as Micronectidae.

## Results

Based on the results of data mining and the Hungarian surveillance monitoring, 58 water bug species representing 21 genera and 12 families are currently known from Hungary (Table 1). The occurence of the species in Hungary is now documented for 37 species of Nepomorpha (Nepidae – 2, Micronectidae – 5, Corixidae – 19, Naucoridae – 1, Aphelocheiridae – 1, Notonectidae – 7, Pleidae – 1) and 21 species of Gerromorpha (Mesoveliidae – 2, Hebridae – 2, Hydrometridae – 2, Veliidae – 6, Gerridae – 9). No representatives of the families Belostomatidae and Ochteridae were found.

**Table 1.** Updated checklist of aquatic and semi-aquatic Heteroptera (Heteroptera: Nepomorpha, Gerromorpha) occurred in Hungary, with the year of the first published occurrence and the author(s).

Taxa	Year of first published occurrence, and author
Nepomorpha	
**Nepidae**	
*Nepa cinerea* Linnaeus, 1758	1918 Horváth
Ranatra (Ranatra) linearis (Linnaeus, 1758)	1918 Horváth
**Micronectidae**	
Micronecta (Dichaetonecta) pusilla (Horváth, 1895)	1918 Horváth
Micronecta (Dichaetonecta) scholtzi (Fieber, 1860)	1918 Horváth
Micronecta (Micronecta) griseola Horváth, 1899	1916 Horváth
Micronecta (Micronecta) minutissima (Linnaeus, 1758)	1962 Wróblewski
Micronecta (Micronecta) poweri poweri (Douglas & Scott, 1869)	1960 Wróblewski
**Corixidae**	
*Cymatia coleoptrata* (Fabricius, 1777)	1885 Horváth
*Cymatia rogenhoferi* (Fieber, 1864)	1885 Horváth
*Callicorixa praeusta praeusta* (Fieber, 1848)	1959 Soós
*Corixa affinis* Leach, 1817	1918 Horváth
*Corixa panzeri* Fieber, 1848	1959 Soós
*Corixa punctata* (Illiger, 1807)	1918 Horváth
*Hesperocorixa linnaei* (Fieber, 1848)	1918 Horváth
*Hesperocorixa sahlbergi* (Fieber, 1848)	1918 Horváth
*Paracorixa concinna concinna* (Fieber, 1848)	1885 Horváth
Sigara (Microsigara) hellensii (C.R. Sahlberg, 1819)	2009 Kiss
Sigara (Pseudovermicorixa) nigrolineata nigrolineata (Fieber, 1848)	1918 Horváth
Sigara (Retrocorixa) limitata limitata (Fieber, 1848)	1918 Horváth
Sigara (Retrocorixa) semistriata (Fieber, 1848)	1918 Horváth
Sigara (Sigara) assimilis (Fieber, 1848)	1959 Soós
Sigara (Sigara) striata (Linnaeus, 1758)	1918 Horváth
*Sigara distincta* (Fieber, 1848)	1918 Horváth
Sigara (Subsigara) falleni (Fieber, 1848)	1918 Horváth
Sigara (Subsigara) fossarum (Leach, 1817)	1990 Bakonyi
Sigara (Vermicorixa) lateralis (Leach, 1818)	1918 Horváth
**Naucoridae**	
*Ilyocoris cimicoides cimicoides* (Linnaeus, 1758)	1918 Horváth
**Aphelocheiridae**	
Aphelocheirus (Aphelocheirus) aestivalis (Fabricius, 1794)	1918 Horváth
**Notonectidae**	
*Anisops sardeus sardeus* Herrich-Schaeffer, 1849	2010 Soós
Notonecta (Notonecta) glauca glauca Linnaeus, 1758	1918 Horváth
Notonecta (Notonecta) lutea Müller, 1776	1918 Horváth
Notonecta (Notonecta) maculata Fabricius, 1794	2009 Soós
Notonecta (Notonecta) meridionalis Poisson, 1926	2009 Soós
Notonecta (Notonecta) viridis Delcourt, 1909	1931 Horváth
Notonecta (Notonecta) obliqua Thunberg, 1787	1938 Visnya
Notonecta (Notonecta) reuteri reuteri Hungerford, 1928	recent paper
**Pleidae**	
*Plea minutissima minutissima* Leach, 1817	1918 Horváth
Gerromorpha	
**Mesoveliidae**	
*Mesovelia furcata* Mulsant et Rey, 1852	1915 Horváth
*Mesovelia thermalis* Horváth, 1915	1999 Kiss
**Hydrometridae**	
*Hydrometra gracilenta* Horváth, 1899	1899 Horváth
*Hydrometra stagnorum* (Linnaeus, 1758)	1878 Horváth
**Hebridae**	
Hebrus (Hebrus) pusillus pusillus (Fallén, 1807)	1878 Horváth
Hebrus (Hebrusella) ruficeps Thomson, 1871	1918 Horváth
**Veliidae**	
Microvelia (Microvelia) buenoi Drake, 1920	1988 Vásárhelyi and Bakonyi
Microvelia (Microvelia) reticulata (Burmeister, 1835)	1916 Horváth
Microvelia (Picaultia) pygmaea (Dufour, 1833)	1916 Horváth
Velia (Plesiovelia) caprai caprai Tamanini, 1947	1923 Horváth
Velia (Plesiovelia) affinis filippii Tamanini, 1947	1938 Visnya
Velia (Plesiovelia) saulii Tamanini, 1947	1969 Benedek
**Gerridae**	
*Aquarius najas* (De Geer, 1773)	1918 Horváth
*Aquarius paludum paludum* Fabricius, 1794	1918 Horváth
Gerris (Gerris) argentatus Schummel, 1832	1878 Horváth
Gerris (Gerris) lacustris (Linnaeus, 1758)	1878 Horváth
Gerris (Gerris) odontogaster (Zetterstedt, 1828)	1918 Horváth
Gerris (Gerris) thoracicus Schummel, 1832	1918 Horváth
Gerris (Gerris) gibbifer Schummel, 1832	1918 Horváth
Gerris (Gerriselloides) asper (Fieber, 1860)	1918 Horváth
*Limnoporus rufoscutellatus* (Latreille, 1807)	1918 Horváth

Although the first checklist listed only 31 species (Horváth 1918), this number increased by 23 species and none disappeared during the time to the second checklist (Kondorosy 1999). From the second checklist to date, the species list has been expanded by five species. Four of these have already been published; *Notonecta maculata* and *Notonecta meridionalis* by Soós et al. (2009), *Anisops sardeus sardeus* by Soós et al. (2010), and *Sigara hellensii* by Kiss et al. (2009), whereas the fifth species, *Notonecta reuteri reuteri* is here recorded for the first from Hungary (see below).

**Figure 1.**
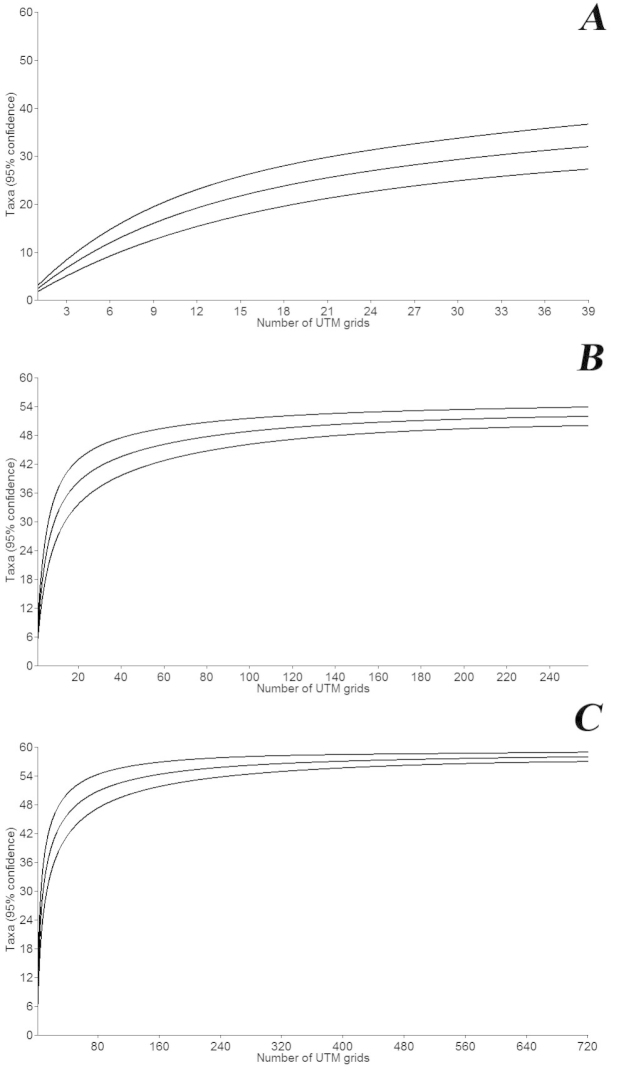
Observed and estimated species richness based on the checklist of given periods. Cumulative species curves produced by PAST 3.02 software package. **A** based on data before the first checklist (published in 1918) **B** based on data before the second checklist (published in 1999) **C** based on the whole database (present work).

Figure 1 represents the species accumulation curves of aquatic and semi-aquatic Heteroptera during the three distinct intervals. The species richness estimators suggest that a large number of species living in the country were not collected before 1918. The estimated number of species was 41, whereas the observed number was only 32. The monotonic increase of the curve confirms that the estimated richness was considerably higher at that time than the observed one. The curve based on data before 1999 showed only a slightly higher estimated taxa richness in Hungary (54) than the observed number of species (52). Based on the most recent (current) checklist, the estimated richness curve flattens off soon after the number of UTM grids increases to 100. The estimated number of species is 58, which is equal to the observed one.

**Figure 2.**
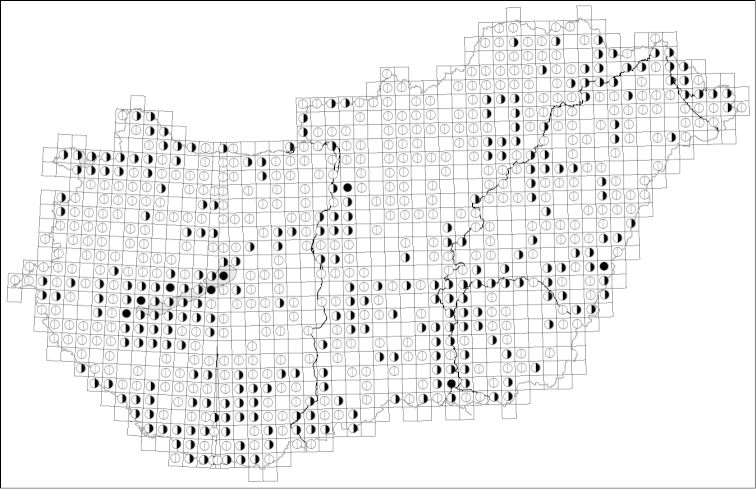
Aggregate records of aquatic and semi-aquatic Heteroptera (Heteroptera: Nepomorpha, Gerromorpha) in Hungary depicted on UTM grids map. Empty circles refer to UTM grids with a lower number of species (*N* < 10), half full circles refer to UTM grids with an average number of species (10 < *N* < 30), and full circles refer to the most diverse UTM grids (*N* > 30).

There are 1061 UTM grid cells in Hungary, 709 of which contain aquatic and semi-aquatic Heteroptera records (66.8% of the total) (Figure 2). The species number in any given grid cell ranged from 0 to 42. The most diverse UTM grid cell was BT70 with 42 species (part of Lake Balaton). Eight grid cells had an outstandingly high number of species (n > 30). Twenty to 30 species occurred in 71 grid cells (10% of the cells in which aquatic and semi-aquatic Heteroptera were found), 10 to 20 species occurred in 204 grid cells (29%), and less than 10 species occurred in 426 grid cells (60%). Finally, there were 352 UTM grid cells without records.

**Figure 3.**
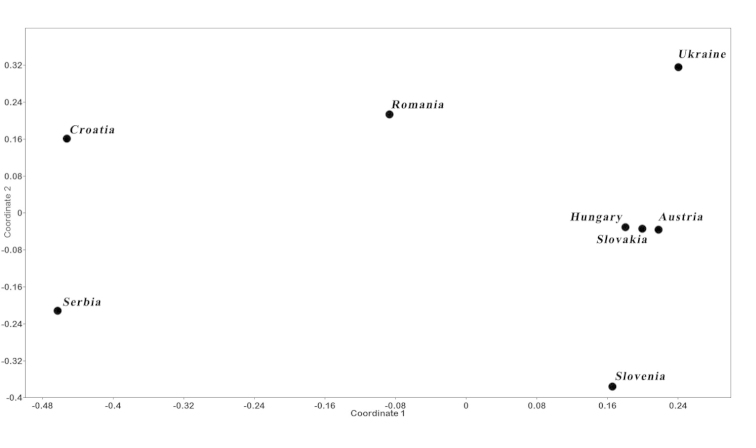
Ordination of the neighbouring countries based on presence-absence data of aquatic and semi-aquatic Heteroptera species (with Jaccard similarity index, Final stress = 0.1998).

The number of species occurring in Hungary (58) corresponds to 36.7% of the water bug fauna of Europe. The number of species was higher in Hungary than in Slovakia (55), Serbia (54), and Slovenia (49); almost the same as in Croatia (59); and slightly lower than in Austria (62), Ukraine (68) and Romania (72) (Table 2, Suppl. material 1). The scatter plot of the NMDS (Figure 3) showed that Hungary had almost the same species list as Slovakia, whereas the other countries surrounding them had slightly different water bug faunas.

**Table 2.** Number of species of aquatic and semi-aquatic Heteroptera from Hungary and neighbouring countries compared to the 158 species in Europe. Data on the number of established species in specific countries taken from different papers.

Countries	Gerromorpha	Nepomorpha	Total number of species	% of the total number of species in Europe
**Slovenia**	20	29	49	31.0
**Slovakia**	20	35	55	34.2
**Serbia**	23	31	54	34.2
**Hungary**	21	37	58	36.7
**Croatia**	22	37	59	37.3
**Austria**	22	40	62	39.2
**Ukraine**	24	44	68	43.0
**Romania**	28	43	72	45.6

### First record of *Notonecta reuteri reuteri*

**Material examined.** *Notonecta reuteri reuteri* Hungerford, 1928: Érd, 1934, 3 females, Pudleiner lgt., P. Boda & P. Kment det. (coll. Hungarian Natural History Museum, Budapest).

Former publications mentioned *Notonecta reuteri reuteri* as a species expected to occur in the Hungarian fauna (Soós et al. 2009, Soós 1963) because it was found in the neighbouring countries. However, it is a tyrphobiont species usually inhabiting higher altitudes in Central Europe (Štys 1960, Wróblewski 1980), i.e., habitats generally absent in Hungary. Recently, 3 females were discovered in the unidentified material of the Hungarian Natural History Museum and were definitively identified as *Notonecta reuteri reuteri*. *Notonecta lutea* and *Notonecta reuteri reuteri* both have the same yellowish scutellum and body shape, but the species are distinguished from each other by the male and female genitalia as well as by the shape of the last abdominal sternum of the female (Štys 1960). There are no recent records of this species from Hungary; it has not been found since 1934, but there is a chance it will be rediscovered in the future. Including *Notonecta reuteri reuteri*, there are now eight species of Notonectidae recorded from Hungary (Soós et al. 2009).

## Discussion

Increased sampling effort contributes to a better knowledge of regional faunas (Dennis et al. 1999, Stander 1998, Rocchini et al. 2011). The number of estimated species in the first period (until 1918) is only a rough estimate due to the small sample size (Figure 1A). It is striking that the small sample size provides a relatively high number of species (Colwell and Coddington 1994). The reason for this lies in how studies were conducted in the beginning of the 20^th^ century. During that period, researchers primarily surveyed the most interesting, particular and diverse habitats. These purposeful and directional studies resulted in the collection of 31 species in a short period of time. More frequent and broadly based studies then yielded higher estimated taxon numbers until 1999. Based on the shape of the species accumulation curve estimated from the entire database until 2013, it appears likely that an increase in sampling efforts will not result in an increase in the number of species currently known from Hungary. Surprisingly, the constantly changing number of studies and the alternating sampling intensity throughout the decades had no traceable influence on the chances of the appearance of a new species. The average rate of species discovery has remained the same, at around 2.85 species per 10 years (23 species in 81 years between 1918 and 1999, and 4 species in 14 years between 2000 and 2014). The constant rate of discovery has no scientific explanation, and can only be considered as a statistical coincidence without any ecological background.

Is the Hungarian aquatic and semiaquatic bug fauna, currently at 58 species, completely known? Our results suggest that the number of species in the country is estimated correctly and that the species accumulation curve levels off at an asymptotic value, a considerable increase in species richness is not expected in the future. It is clear that species composition may change and that the opportunity of species turnover exists. Turnover of species, or finding additional species new to Hungary, depends on the current characteristics of water bodies and on the biological attributes controlling the dispersal and persistence of their potential colonists (Case and Cody 1987). In former publications, 24 species were considered as expected species on Hungarian fauna (Soós 1963, Benedek 1969). Six of these species are now confirmed members of the fauna, and the others might appear in the future. What a species needs and what the environment supplies is species-specific, but due to the fact that the borders of several eco-regions meet in the Carpathian Basin, Mediterranean, and Eurosiberian species occur along with Holarctic and Palaearctic species (Josifov 1986). Because of this biogeographic setting, the chance for the appearance of additional species is difficult to predict accurately.

Among these expected species, some alien species show a recent range expansion northwards in Europe (Van de Meutte et al. 2010, Boda et al. 2012, Guareschi et al. 2013, Barbora and Marek 2014, Reduciendo Klementová and Svitok 2014). Several new records and regular findings of *Anisops sardeus sardeus* were published from all across Europe during the last five years (Berchi 2011, Khatukhov et al. 2011, Kment and Beran 2011, Cianferoni and Pinna 2012, Cianferoni and Terzani 2013, Reduciendo Klementová and Svitok 2014) and from Hungary (Soós et al. 2010). In addition, Hungary is a potential area of invasion of another alien bug *Trichocorixa verticalis verticalis* (Fieber, 1851) (Corixidae). The possibility of the future occurrence of this taxon is high for several reasons. First, this species lives in brackish and saline waters in both juvenile and adult phases, salinity tolerance is one of the key factors for its expanding range (Van De Meutter et al. 2010), and the Carpathian Basin is extremely rich in soda pans. Second, climate change is generally expected to result in increased salinization of water bodies. Finally, the resting eggs of this species are able to survive in extreme environments (Tones 1977, Kelts 1979). These facts together can facilitate the appearance of this species and the survival of the pioneer individuals in Hungary (Guareschi et al. 2013).

The national biodiversity monitoring system of Hungary is operated at approximately 1200 samplings stations from 558 UTM grid cells and thus provides a broad spatial coverage. With the addition of UTM grid cells where further studies were carried out with various purposes and which provided valid data (198 papers altogether), the spatial coverage has now reached two thirds of the area of Hungary. The most diverse grid cells may have particular significance for biodiversity conservation as hotspots of species richness. However, the eight grids with an outstandingly high number of species (*N* > 30) can also result from unusually high sampling effort. Five from the eight cells belong to Lake Balaton and its tributaries, one of the most frequently studied shallow lakes in Europe (BT70: Horváth 1931, Bakonyi and Vásárhelyi 1988, Bíró and Hufnagel 1998, 2001, Bíró 2003, Sipkay et al. 2005, Vásárhelyi and Bakonyi 2005, 2012; XM67: Soós 1959, Kondorosy et al. 1996, 2011, Bíró and Hufnagel 1998, Kiss et al. 2008, Móra et al. 2008; XM78: Horváth 1931, Soós 1959, Wróblewski 1960, Bíró and Hufnagel 1998, Kiss et al. 2008, Kondorosy 2011; XM99: Soós 1959, Bíró and Hufnagel 1998, Rozner 2004, Móra et al. 2007, 2011, Szekeres and Csányi 2010; and YM29: Horváth 1931, Soós 1959, Wróblewski 1960, Bíró and Hufnagel 1998, Móra et al. 2007, Kiss et al. 2008, Soós et al. 2009). Grid cells with similarly high richness also occur near Szeged, at the site of a periodic and long-term study (DS32: Vellay 1899, Czógler 1937, Csongor 1956, Soós 1959, Csabai et al. 2010); near Budapest, at the site of a continuous but medium-term (1991–1996) ecological study (CT66: Hufnagel 1994, 1998); and Kis‐Sárrét Nature Conservation area (SE, Hungary), at the site of an intensive but short-term study with several sampling times per year (ET40: unpublished personal data). These considerations suggest that these regions are not necessarily hotspots of species richness, they rather reflect a disproportionately high sampling effort in these grid cells. On the other hand, the UTM grid cells with no records show a random and patchy pattern. Surveys in these UTM grids provide some chance for the appearance of species new to the country.

A comparison of species composition with that of neighbouring countries is difficult because of the high variation in latitude, area, climate, altitude, and the number and types of watercourses. In Hungary, all but one catchment area originates in the surrounding mountain ranges (the Alps to the west, Carpathians to the north and east, and Dinarids to the south) and thus extends beyond the country borders. As a result, drift phenomena from upstream reaches can be more frequent and important than one might think. No species occurs exclusively in Hungary, which could be explained by these geographical features, the fact that the country borders are not aligned with any geographical feature and that aquatic bugs have good dispersal abilities. Dispersal studies indicate that 32% of the fauna can be found in the air as common species (Csabai et al. 2012, Boda and Csabai 2013, Boda et al. 2014). On the other hand, the species/area relationship suggests that the number of species in an area correlates strongly and positively with the size of that area. In the last decade, specialists in neighbouring countries made a considerable effort to explore the aquatic and semi-aquatic Heteroptera fauna (Austria: Rabitsch 2008a,b; Croatia: Kment and Beran 2011, Turić et al. 2011; Romania: Berchi 2011, 2013, Berchi et al. 2011, 2012, Ilie and Olosutean 2012; Serbia: Živić et al. 2007, Šeat 2011, 2013, Protić 2011, Protić and Živić 2012; Slovakia: Klementová et al. 2012, Kment et al. 2013, Reduciendo Klementová and Svitok 2014; Slovenia: Gogala 2003, 2009; Ukraine (including Crimea): Putshkov and Putshkov 1996, Grandova and Prokin 2012, Grandova 2013, 2014). Consequently, the aquatic and semi-aquatic Heteroptera fauna of these countries is adequately known, except for Ukraine, the large area of which sets a natural limit to the number of surveys. In our case, there is a strong positive correlation between the number of species and the area of the countries (r = 0.695, n = 8, p < 0.05). Moreover, the correlation coefficient is even higher and significant (r = 0.905, n = 7, p < 0.05) with Ukraine excluded from the analysis because of its under-studied status.

The plot of the NMDS and the geographical map has shown the same organizing principles. Hungary and Slovakia together are roughly at the same latitude with Austria and two other countries with similar geographical/environmental conditions (Romania, Ukraine), whereas countries reaching into the Mediterranean Region are located further south (Croatia, Serbia, Slovenia). The differences in faunal composition seen in the plot should be due to the rare or unique species, and 33 species are common in the faunas of the eight countries (Suppl. material 1). It is well known that latitude has a major influence on species diversity (Fischer 1960) with species richness increasing from high latitudes toward the tropics (Rosenzweig 1995). The latitudinal pattern of aquatic bugs is currently unknown, and has been rarely studied for the whole macroinvertebrate community. Our data suggests that there is no evidence for such a latitudinal diversity gradient at our spatial scale. However, our data confirm that latitude *per se* cannot be a determinant of species richness; diversity only correlates with a number of potentially causal environmental factors (Gaston 2000). Even if species richness does not show correlation with latitude, similar species compositions were observed in the countries positioned along the same latitude. We found three main groups based on species number and fauna composition: (1) slightly lower number of species, but unique fauna composition, e.g., Slovenia, Serbia and Croatia; (2) average number of species, with highly overlapping fauna composition, e.g., Hungary, Slovakia and Austria; (3) higher number of species with many species in common with countries in group 2 along with some extra species occurring in larger and more heterogeneous countries (Romania, Ukraine).

We conclude that the species list and the UTM-based database are now up-to-date for Hungary. These will provide a basis for future studies of distributional and biodiversity patterns, biogeography, relative abundances and frequency of occurrences important in community ecology, or the determination of conservation status.

## Supplementary materials

Supplementary material 1 Checklist of aquatic and semi-aquatic Heteroptera (Heteroptera: Nepomorpha, Gerromorpha) occurred in Hungary, and the neighbouring countries Data type: occurence data This dataset is made available under the Open Database License (http://opendatacommons.org/licenses/odbl/1.0/). The Open Database License (ODbL) is a license agreement intended to allow users to freely share, modify, and use this Dataset while maintaining this same freedom for others, provided that the original source and author(s) are credited.Pál Boda, Tamás Bozóki, Tamás Vásárhelyi, Gábor Bakonyi, Gábor Várbíró
