# Supplementary material for: Revised and annotated checklist of aquatic and semi-aquatic Heteroptera of Hungary with comments on biodiversity patterns
Source: Zookeys. 2015 Apr 30;(501):89–108. doi: 10.3897/zookeys.501.8964 (PMC4432320; doi:10.3897/zookeys.501.8964)
Supplement: Supplementary material 1 — Checklist of aquatic and semi-aquatic Heteroptera (Heteroptera: Nepomorpha, Gerromorpha) occurred in Hungary, and the neighbouring countries [file zookeys-501-089-s001.docx]

**Supplementary files**

**for**

**Revised and annotated checklist of aquatic and semi-aquatic Heteroptera of Hungary with comments on biodiversity patterns**

Pál Boda^1*^– Tamás Bozóki^2^ – Tamás Vásárhelyi^3^ – Gábor Bakonyi^4^ – Gábor Várbíró^1^

^1^MTA Centre for Ecological Research, Department of Tisza River Research, Bem tér 18/c., H-4026 Debrecen, Hungary

^2^Eszterházy Károly College, Eszterházy tér 1., H‐3300, Eger, Hungary

^3^Hungarian Natural History Museum, Baross u. 13., H-1088, Budapest, Hungary

^4^Szent István University, Department of Zoology and Animal Ecology, Páter Károly u. 1., H-2100, Gödöllő, Hungary

*corresponding author, e-mail address: [boda.pal@okologia.mta.hu](mailto:boda.pal@okologia.mta.hu)

**This file includes the following: Supplementary Table S1**

Table S1: Checklist of aquatic and semi-aquatic Heteroptera (Heteroptera: Nepomorpha, Gerromorpha) occurred in Hungary, and the neighbouring countries.

| **Species** | **Austria** | **Croatia** | **Hungary** | **Romania** | **Serbia** | **Slovakia** | **Slovenia** | **Ukraine** |
| --- | --- | --- | --- | --- | --- | --- | --- | --- |
| **Nepomorpha** |  |  |  |  |  |  |  |  |
| **Nepidae** |  |  |  |  |  |  |  |  |
| *Nepa anophthalma* Decu, Gruia, Keffer & Sarbu, 1994 | - | - | - | + | - | - | - | - |
| *Nepa cinerea* Linnaeus, 1758 | + | + | + | + | + | + | + | + |
| *Ranatra linearis* (Linnaeus, 1758) | + | + | + | + | + | + | + | + |
| [**Belostomatidae**](http://en.wikipedia.org/wiki/Belostomatidae) |  |  |  |  |  |  |  |  |
| *Lethocerus patruelis* (Stål, 1854) | - | + | - | + | + | - | - | - |
| **Ochteridae** |  |  |  |  |  |  |  |  |
| *Ochterus marginatus marginatus* (Latreille, 1804) | - | + | - | - | - | - | - | - |
| **Micronectidae** |  |  |  |  |  |  |  |  |
| *Micronecta pusilla*(Horváth,, 1895) | - | + | + | + | + | - | - | + |
| *Micronecta scholtzi*(Fieber,, 1860) | + | + | + | + | + | + | + | + |
| *Micronecta carpatica*Wróblewski, 1958 | - | - | - | - | + | + | - | - |
| *Micronecta griseola*Horváth, 1899 | + | - | + | + | - | + | + | + |
| *Micronecta minutissima*(Linnaeus,, 1758) | - | - | + | + | + | + | - | + |
| *Micronecta poweri poweri*(Douglas & Scott, 1869) | + | - | + | + | - | + | + | + |
| **Corixidae** |  |  |  |  |  |  |  |  |
| *Arctocorisa carinata carinata*(C.R. Sahlberg,, 1819) | + | - | - | + | + | - | + | - |
| *Arctocorisa germari*(Fieber, 1848) | + | + | - | - | - | - | - | - |
| *Callicorixa praeusta praeusta*(Fieber, 1848) | + | - | + | + | - | + | - | + |
| *Corixa affinis*Leach, 1817 | + | + | + | + | + | + | + | + |
| *Corixa dentipes*Thomson, 1869 | + | - | - | + | - | - | - | + |
| *Corixa panzeri*Fieber, 1848 | + | + | + | + | + | + | - | + |
| *Corixa punctata*(Illiger, 1807) | + | + | + | + | + | + | + | + |
| *Cymatia bonsdorffii*(C.R. Sahlberg, 1819) | + | - | - | + | - | - | - | + |
| *Cymatia coleoptrata*(Fabricius, 1777) | + | + | + | + | + | + | + | + |
| *Cymatia rogenhoferi*(Fieber, 1864) | + | - | + | + | + | + | - | + |
| *Glaenocorisa propinqua*(Fieber, 1860) | + | + | - | + | - | + | - | + |
| *Hesperocorixa linnaei*(Fieber, 1848) | + | + | + | + | + | + | + | + |
| *Hesperocorixa moesta*(Fieber, 1848) | + | + | - | - | - | + | - | + |
| *Hesperocorixa parallela*(Fieber, 1860) | - | + | - | + | - | - | + | + |
| *Hesperocorixa Sahlberg,i*(Fieber, 1848) | + | + | + | + | + | + | + | + |
| *Paracorixa concinna concinna*(Fieber, 1848) | + | + | + | + | + | + | + | + |
| *Sigara mayri*(Fieber, 1860) | - | - | - | + | - | - | - | + |
| *Sigara stagnalis pontica*Jaczewski, 1961 | - | + | - | + | - | - | - | + |
| *Sigara hellensii*(C.R. Sahlberg, 1819) | + | - | + | - | - | - | + | + |
| *Sigara nigrolineata nigrolineata*(Fieber, 1848) | + | + | + | + | + | + | + | + |
| *Sigara limitata limitata*(Fieber, 1848) | + | + | + | + | + | + | + | + |
| *Sigara semistriata*(Fieber, 1848) | + | + | + | + | + | + | + | + |
| *Sigara assimilis*(Fieber, 1848) | - | + | + | + | - | - | - | + |
| *Sigara dorsalis*(Leach, 1817) | - | - | - | - | + | - | - | - |
| *Sigara striata*(Linnaeus, 1758) | + | + | + | + | + | + | + | + |
| *Sigara distincta*(Fieber, 1848) | + | - | + | - | - | - | + | + |
| *Sigara falleni*(Fieber, 1848) | + | + | + | + | + | + | + | + |
| *Sigara fossarum*(Leach, 1817) | + | + | + | + | - | + | + | + |
| *Sigara iactans*Jansson, 1983 | - | - | - | + | + | - | - | + |
| *Sigara longipalis*(J. Sahlberg, 1878) | + | - | - | - | - | - | - | + |
| *Sigara scotti*(Douglas & Scott, 1868) | - | - | - | - | - | - | - | + |
| *Sigara lateralis*(Leach, 1817) | + | + | + | + | + | + | + | + |
| *Sigara scripta*(Rambur, 1840) | - | + | - | - | - | - | - | - |
| **Naucoridae** |  |  |  |  |  |  |  |  |
| *Ilyocoris cimicoides cimicoides* (Linnaeus, 1758) | + | + | + | + | + | + | + | + |
| **Aphelocheiridae** |  |  |  |  |  |  |  |  |
| *Aphelocheirus aestivalis* (Fabricius, 1794) | + | + | + | + | + | + | + | + |
| **Notonectidae** |  |  |  |  |  |  |  |  |
| *Anisops sardeus sardeus* Herrich-Schäffer, 1849 | - | + | + | + | - | + | - | - |
| *Notonecta glauca glauca* Linnaeus, 1758 | + | + | + | + | + | + | + | + |
| *Notonecta lutea* Müller, 1776 | + | + | + | + | + | + | - | + |
| *Notonecta maculata* Fabricius, 1794 | + | + | + | + | - | + | + | - |
| *Notonecta meridionalis* Poisson, 1926 | + | + | + | + | - | - | + | + |
| *Notonecta obliqua* Thunberg, 1787 | + | + | + | - | + | + | - | - |
| *Notonecta reuteri reuteri* Hungerford, 1928 | + | - | + | - | - | + | - | + |
| *Notonecta viridis* Delcourt, 1909 | + | + | + | + | + | + | + | + |
| **Pleidae** |  |  |  |  |  |  |  |  |
| *Plea minutissima minutissima* Leach, 1817 | + | + | + | + | + | + | + | + |
| **Gerromorpha** |  |  |  |  |  |  |  |  |
| **Mesoveliidae** |  |  |  |  |  |  |  |  |
| *Mesovelia furcata* Mulsant & Rey, 1852 | + | + | + | + | + | + | + | + |
| *Mesovelia thermalis* Horváth, 1915 | - | - | + | + | - | - | - | + |
| *Mesovelia vittigera* Horváth, 1895 | - | + | - | + | + | - | - | - |
| **Hydrometridae** |  |  |  |  |  |  |  |  |
| *Hydrometra gracilenta* Horváth, 1899 | + | - | + | + | - | + | - | + |
| *Hydrometra stagnorum* (Linnaeus, 1758) | + | + | + | + | + | + | + | + |
| **Hebridae** |  |  |  |  |  |  |  |  |
| *Hebrus fulvineris* Horváth, 1929 | - | - | - | + | - | - | - | - |
| *Hebrus montanus* Kolenati, 1857 | - | - | - | + | + | + | - | - |
| *Hebrus pilipes* Kanyukova, 1997 | - | - | - | - | - | - | - | - |
| *Hebrus pusillus pusillus* (Fallen, 1807) | + | + | + | + | + | + | + | + |
| *Hebrus ruficeps* Thomson, 1871 | + | + | + | + | + | + | + | + |
| **Veliidae** |  |  |  |  |  |  |  |  |
| *Microvelia buenoi* Drake, 1920 | + | - | + | - | - | + | - | + |
| *Microvelia reticulata* (Burmeister, 1835) | + | + | + | + | + | + | + | + |
| *Microvelia pygmaea* (Dufour, 1833) | + | + | + | + | + | - | + | - |
| *Velia affinis affinis* Kolenati, 1857 | - | - | - | - | - | - | - | + |
| *Velia affinis filippii* Tamanini, 1947 | - | + | + | + | + | - | + | - |
| *Velia caprai caprai* Tamanini, 1947 | + | - | + | + | + | + | + | + |
| *Velia currens* (Fabricius, 1794) | + | + | - | + | + | - | + | - |
| *Velia mancinii mancinii* Tamanini, 1947 | - | + | - | + | - | - | - | - |
| *Velia muelleri* Tamanini, 1947 | - | + | - | - | - | - | - | - |
| *Velia pelagonensis* Hoberlandt, 1941 | - | + | - | - | + | - | - | - |
| *Velia saulii* Tamanini, 1947 | + | - | + | + | - | + | + | + |
| *Velia serbica* Tamanini, 1951 | - | - | - | - | + | - | - | - |
| *Velia rivulorum* (Fabricius, 1775) | - | - | - | + | - | - | - | - |
| **Gerridae** |  |  |  |  |  |  |  |  |
| *Aquarius najas* (De Geer, 1773) | + | + | + | + | + | + | + | + |
| *Aquarius paludum paludum* (Fabricius, 1794) | + | + | + | + | + | + | + | + |
| *Gerris argentatus* Schummel, 1832 | + | + | + | + | + | + | + | + |
| *Gerris costae costae* (Herrich-Schäffer 1850) | + | - | - | - | - | - | + | - |
| *Gerris costae fieberi* Stichel, 1938 | - | + | - | + | + | - | - | + |
| *Gerris gibbifer* Schummel, 1832 | + | + | + | + | + | + | + | + |
| *Gerris lacustris* (Linnaeus, 1758) | + | + | + | + | + | + | + | + |
| *Gerris maculatus* Tamanini, 1946 | - | + | - | + | - | - | - | + |
| *Gerris odontogaster* (Zetterstedt, 1828) | + | + | + | + | + | + | + | + |
| *Gerris sphagnetorum* Gaunitz, 1947 | - | - | - | - | - | - | - | + |
| *Gerris thoracicus* Schummel, 1832 | + | + | + | + | + | + | + | + |
| *Gerris asper* (Fieber, 1860) | + | + | + | + | + | + | + | + |
| *Gerris lateralis* Schummel, 1832 | + | - | - | + | + | + | - | + |
| *Limnoporus rufoscutellatus* (Latreille, 1807) | + | - | + | + | - | + | + | + |
| ***Total number of species*** | **62** | **59** | **58** | **72** | **54** | **55** | **49** | **68** |
